# Conformable Endovascular Aneurysm Repair (EVAR) Stability in Hostile Neck Abdominal Aortic Aneurysms: A Single-Center Experience

**DOI:** 10.7759/cureus.95395

**Published:** 2025-10-25

**Authors:** Ayman Zyada, Rutwik Hegde, Mohamed Eltayeb Abdelrahman Naiem, Bryce Renwick

**Affiliations:** 1 Vascular Surgery, Aberdeen Royal Infirmary, Aberdeen, GBR

**Keywords:** abdominal aortic aneurysm, aortic endograft stability, endovascular aneurysm repair, endovascular reintervention, gore excluder conformable, hostile neck anatomy, proximal fixation, stent graft migration

## Abstract

Background

Stent graft migration is a well-recognized complication of endovascular aneurysm repair (EVAR) that can predispose patients to type I endoleaks, renal artery compromise, and secondary interventions, particularly in cases with hostile neck anatomy, which poses a significant technical challenge for graft fixation. The GORE Excluder Conformable AAA Endoprosthesis (CLEVAR) is specifically designed to address these challenges by accommodating neck lengths as short as 10 mm, diameters down to 16 mm, and angulations up to 90°. This study aimed to evaluate the early stability and safety of the CLEVAR in patients with hostile neck anatomy by assessing its migration behavior within the first postoperative year.

Methodology

This retrospective, single-center study analyzed consecutive patients who underwent repair of hostile neck abdominal aortic aneurysms with the CLEVAR device between January 2019 and December 2022, focusing on early (≤1 year) postoperative stability. Eligible patients completed both three-month and one-year postoperative CT angiography. Migration was assessed by measuring the distance between the proximal stent edge and the bilateral renal arteries at both time points, with the difference representing stent movement. Descriptive statistics were calculated, normality was tested using the Shapiro-Wilk method, and comparisons were performed with nonparametric Wilcoxon signed-rank tests.

Results

A total of 25 patients were included. The mean stent migration across the cohort was 1.29 mm (SD 1.36 mm), with a median of 0.85 mm and a maximum migration of 6.0 mm. Both right and left renal reference points showed similar results (mean 1.17 mm and 1.40 mm, respectively), with no statistically significant difference between sides (p = 0.4261). Migration distributions were non-normally distributed and positively skewed. Analysis confirmed that stent displacement was statistically greater than zero (p < 0.00000001), indicating measurable, albeit small, positional changes during the interval.

Conclusions

In this study, the CLEVAR device demonstrated stable proximal fixation in challenging hostile neck anatomies over the first postoperative year. Observed migration was minimal, symmetrical, and well below thresholds considered clinically significant. These findings suggest that CLEVAR provides reliable early stability in anatomically complex aneurysm necks, potentially reducing the risk of migration-associated complications. However, the small sample size, retrospective design, and one-year follow-up limit generalizability. Longer-term studies with larger cohorts are needed to confirm the durability of fixation and explore the relationship between late migration, endoleak incidence, and the need for reintervention.

## Introduction

Stent migration is a notable complication following endovascular aneurysm repair (EVAR) that can lead to type I endoleak or renal artery occlusion, with multiple factors influencing its incidence and severity. In a study of abdominal aortic aneurysms (AAAs), 56% of patients experienced migration of ≥4 mm, with significant associations observed between migration and nonadherence to device instructions for use [1]. Structural failures of specific devices have also been implicated in migration cases, underscoring the need for careful postoperative monitoring. Another study reported significant stent graft movement at a threshold of 9 mm, with migration occurring in 31.5% of patients [2]. Additionally, a review of commercially available devices found a migration rate of 1.8% [3]. In cases involving large proximal necks, migration has been defined as a positional change exceeding 1 cm [4].

One of the most important anatomical factors that exacerbates stent migration complications, and is associated with increased perioperative risks and reintervention rates, is hostile neck anatomy. In AAAs, hostile neck anatomy is defined by characteristics such as angulation >60°, diameter >28 mm, or length <15 mm. Hostile necks challenge standard repair techniques, often necessitating alternative approaches or specialized devices [5]. One such device is the GORE Excluder Conformable AAA Endoprosthesis (CLEVAR), developed by W. L. Gore & Associates (Newark, DE, USA). This device is approved for use in hostile necks with lengths as short as 10 mm, neck angulations up to 90°, and diameters as small as 16 mm [6-8].

The aim of this study was to assess the migration potential in EVAR cases treated with the CLEVAR.

## Materials and methods

This retrospective, single-center study included all consecutive patients who underwent EVAR using the CLEVAR device between January 2019 and December 2022 for AAAs with hostile neck anatomy. Hostile neck anatomy was defined according to the criteria of the Society for Vascular Surgery (SVS), including a proximal neck length of less than 15 mm, angulation greater than 60°, significant circumferential calcification, or the presence of mural thrombus.

Eligible patients were those who underwent EVAR with the CLEVAR device and had follow-up contrast-enhanced CT angiography (CTA) performed at both three months and one year postoperatively. Only patients with imaging of adequate quality permitting reliable measurement of stent position in relation to the renal arteries were included. Patients were excluded if they had been treated with a device other than CLEVAR, if CTA was unavailable at one or both follow-up time points, or if the device had been explanted before completion of the one-year follow-up. CTA studies were considered of adequate quality when the axial slice thickness was ≤1.25 mm and arterial phase opacification provided clear visualization of the aortic neck and renal arteries.

All CTA examinations were reviewed retrospectively by two independent observers. For each patient, the distance between the proximal edge of the stent graft and the ostium of each renal artery was measured on both the three-month and one-year studies. Migration distance was defined as the difference in these measurements between the two time points. This yielded two migration values per patient, one for the right renal artery and one for the left.

Statistical analysis was performed to evaluate both overall migration and potential differences between the two sides. The normality of the migration data was assessed using the Shapiro-Wilk test. As the distribution of values for both renal arteries deviated from normality, the Wilcoxon signed-rank test was applied to compare right- and left-sided migration. Descriptive statistics were reported as mean ± SD for continuous variables, while categorical data were expressed as counts and percentages. Migration distances were further illustrated graphically using a histogram and a box plot to depict overall distribution and variability. All statistical analyses were conducted using Stata version 12.0 (StataCorp LLC, College Station, TX, USA).

## Results

A total of 25 patients were included in the study. Table 1 summarizes their demographic characteristics.

**Table 1:** Demographic characteristics of the study cohort

Variable	Value
Number of patients	25
Age, mean ± SD	79.2 ± 6.3 years
Age, median (IQR)	80.0 (73.0-83.0)
Male sex, n (%)	20 (80.0%)
Female sex, n (%)	5 (20.0%)

We analyzed whether there was a significant difference in migration distance between the right and left renal arteries. The results are summarized in Table 2.

**Table 2:** Analysis of stent migration distance from the right and left renal arteries

Metric	Right side	Left side
Mean	1.172 mm	1.404 mm
SD	1.34 mm	1.43 mm
Min	0.0 mm	0.0 mm
25th percentile	0.2 mm	0.4 mm
Median	0.7 mm	0.9 mm

Normality of the data was assessed using the Shapiro-Wilk test. Both the right-sided measurements (p = 0.0002) and the left-sided measurements (p = 0.0011) significantly deviated from a normal distribution. Consequently, a nonparametric test was applied for paired comparison. The Wilcoxon signed-rank test was used to evaluate differences in stent graft migration between the right and left renal artery reference points. The analysis yielded a test statistic of 132.5 with a corresponding p-value of 0.4261, indicating that there was no statistically significant difference in migration distance between the two sides at the 5% significance level.

**Figure 1:**
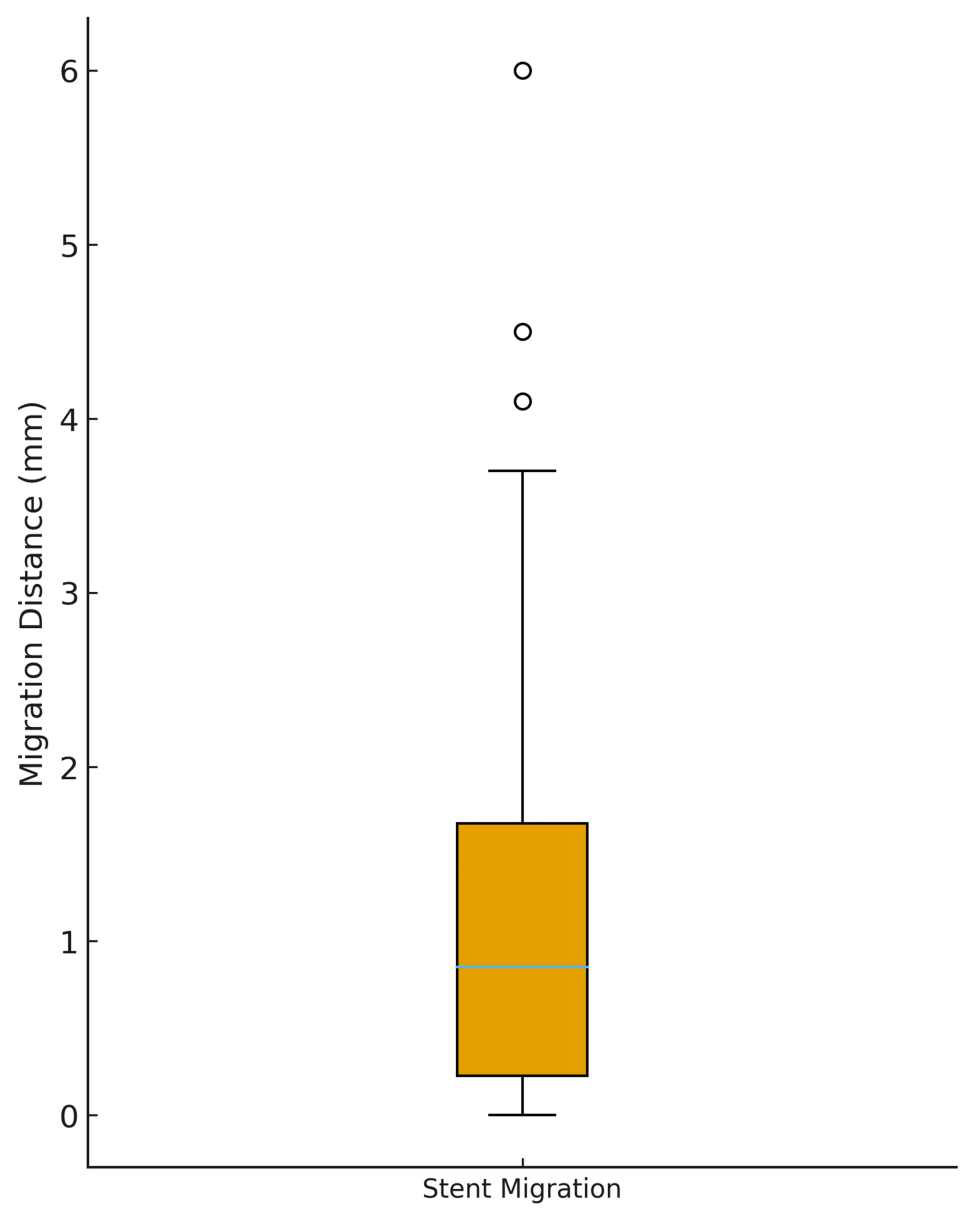
Box plot of stent graft migration distances The box plot shows stent migration distances between the three-month and one-year postoperative CT angiograms. The median migration was approximately 1 mm, with most values clustering below 2 mm. Outliers up to 6 mm were observed.

**Figure 2:**
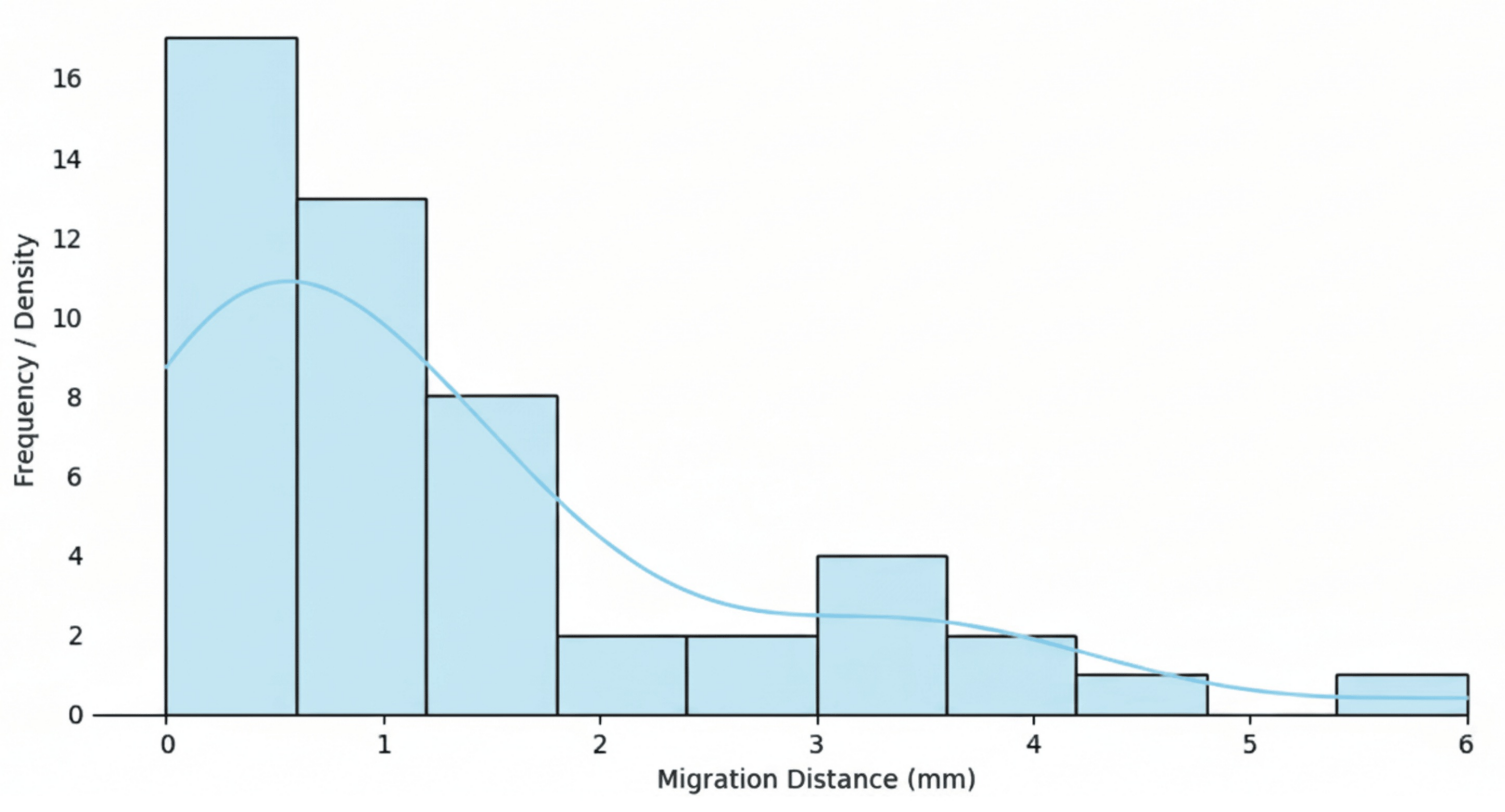
Distribution of migration distances Histogram illustrating the distribution of stent migration distances between the three-month and one-year postoperative CT angiograms. Most migration measurements clustered between 0 and 2 mm, with a few higher values up to 6 mm, indicating that the majority of cases demonstrated minimal displacement.

Analysis of overall stent graft migration between the three-month and one-year postoperative CTAs demonstrated a mean migration distance of 1.29 mm (SD, 1.36 mm). The median migration distance was 0.85 mm, with an IQR of 1.45 mm. The minimum observed migration was 0 mm, and the maximum was 6.0 mm, indicating the presence of a small number of higher-value outliers within the cohort. These results are illustrated in Figure 1 (box plot) and Figure 2 (distribution histogram).

Cumulative distribution function

This plot shows the probability that a stent migrated up to a certain distance. For example, approximately 60% of stents migrated ≤1.5 mm, and about 80% migrated ≤2.5 mm.

Assessment of the distribution of migration values using the Shapiro-Wilk test demonstrated significant deviation from normality (W = 0.83, p < 0.00001), confirming that the data were not normally distributed and were positively skewed.

To determine whether stent migration differed significantly from zero, the Wilcoxon signed-rank test was applied. The analysis demonstrated a highly significant result (test statistic = 0.0, p < 0.00000001), indicating that median stent migration was greater than zero and that measurable displacement occurred between the three-month and one-year intervals. This finding suggests that, although small stent movements were common, clinically significant migration was rare in this study population.

## Discussion

In this retrospective analysis, we evaluated stent graft migration in patients who underwent AAA repair with the CLEVAR device for hostile neck anatomy, focusing on interval changes between the three-month and one-year postoperative CT angiograms. Our findings demonstrated a mean migration distance of 1.29 mm (SD, 1.36 mm), and importantly, no statistically significant asymmetry was detected between the right and left renal artery reference points. These results suggest that, within the first postoperative year, stent migration remains minimal and largely symmetrical, even in anatomically challenging cases.

Stent graft migration is a well-recognized concern following EVAR, particularly in patients with hostile neck anatomy, as it predisposes them to type I endoleaks, device failure, and secondary interventions [1]. According to the SVS, clinically relevant stent migration is typically defined as a downward displacement of more than 10 mm in the proximal landing zone [1]. In comparison, the migration distances observed in our cohort were small, suggesting adequate proximal fixation during the first year after implantation. This favorable stability may reflect the anchoring design of the CLEVAR device.

The absence of significant differences between right- and left-sided renal measurements further suggests uniform proximal fixation, supporting the reliability of CLEVAR across both renal ostia. This finding is clinically relevant because asymmetric migration could compromise renal perfusion and contribute to functional decline. Our results align with prior reports on the device. For instance, a 2023 study reported migration >10 mm in 4.3% of patients during a median follow-up of 10 months [9]. Similarly, a smaller series of 20 patients treated with CLEVAR identified one type I endoleak (5%) but did not report migration distances [10]. Together, these data indicate that while minor positional changes are common, clinically significant migration is relatively uncommon in the early postoperative period.

Several limitations of our study must be acknowledged. The sample size was modest (25 patients and 50 renal artery measurements), and the retrospective design limits generalizability. In addition, our analysis relied on renal artery landmarks to assess migration, which may not capture other forms of device movement, such as distal migration or changes in angulation. The absence of a comparator group further limits device-specific conclusions. Finally, the one-year follow-up period is insufficient to evaluate long-term stability, as migration may occur several years after EVAR.

Despite these limitations, our findings contribute to the growing body of evidence suggesting that the CLEVAR device provides stable proximal fixation in patients with hostile neck anatomy during the first year after implantation [11-13]. Longer-term studies with larger cohorts are warranted to determine whether this early stability persists beyond 12 months and whether late migration is associated with adverse outcomes such as type I endoleaks, device failure, or the need for secondary intervention.

## Conclusions

In patients with hostile neck anatomy treated with the CLEVAR device, stent graft migration during the first postoperative year was minimal, symmetrical, and below the threshold generally considered clinically significant. These findings support the early stability of CLEVAR fixation; however, longer-term surveillance and larger studies are required to confirm its durability and to clarify the relationship between late migration, endoleak development, and the need for reintervention.
